# Supplementary material for: A multi-state model of the CaMKII dodecamer suggests a role for calmodulin in maintenance of autophosphorylation
Source: PLoS Comput Biol. 2019 Dec 23;15(12):e1006941. doi: 10.1371/journal.pcbi.1006941 (PMC6957207; doi:10.1371/journal.pcbi.1006941)
Supplement: S1 Appendix — (DOCX) [file pcbi.1006941.s001.docx]

**Appendix S1**

Supplemental Material:

A Multi-State Model of the CaMKII Dodecamer Suggests

a Role for Calmodulin in Maintenance of Autophosphorylation

Matthew C. Pharris, Neal M. Patel, Tyler G. VanDyk, Thomas M. Bartol, Terrence J. Sejnowski, Mary B. Kennedy, Melanie I. Stefan, and Tamara L. Kinzer-Ursem

Contents:

Table A. Enumeration of model state transitions, rates parameters, conditions, and citations.

Table B. Transitions, rates parameters, conditions, and citations for the non-exclusive model.

Table C. Transitions, rates parameters, conditions, and citations for the 9-state-1-step model.

Figure A. Model validation at long Ca^2+^ peak widths.

Figure B. CaM activation/inactivation parameters as a function of time.

Text A. Quantification of 12-subunit combinatorial explosion by necklace numbers.

Figure C. Alternative presentation of Fig 4 (over a two-second time window).

Text B. Exploration of multiple degrees of freedom for Thr-286 auto-phosphorylation.

Figure D. Schematic of higher-DoF autophosphorylation of CaMKII subunits.

Figure E. Comparing pThr-286 formation for various model DoF for autophosphorylation.

Figure F. Monitoring individual CaMKII subunits at distinct autophosphorylation DoF

Figure G. pThr-286 chain occurrence increases with increasing DoF.

Movie A. Visualization of subunit tracking multiple states on the same simulated holoenzyme.

Text C. Discussion of putative PP1-CaMKII binding site.

Figure H. Putative binding interaction between spinophilin and PP1.

Figure I. CaMKII subunit domains.

Figure J. Putative PP1-binding site on CaMKII regulatory domain.

Figure K. Major Ca^2+^/CaM-CaMKII states following Ca^2+^ bolus in the 9-state-1-step model

Figure L. Ca^2+^/CaM-CaMKII states with dynamic Ca^2+^ stimulation and the 9-state-1-step model

References (Supplement)

**Table A. CaMKII model state transitions, grouped by flag, with rates and conditions provided.**

| Flag | State Transition | Rate | Condition (Rule) | References |
| --- | --- | --- | --- | --- |
| 1. Docking | Docked 🡪 Undocked | 0.63 s^-1^ | Subunit Flag 1 == Docked | [1-3], this paper |
|  | Undocked 🡪 Docked | 35 s^-1^ | Subunit Flag 3 == Unbound  Subunit Flag 5 == uThr-306 | [1-3], this paper |
| 1. Activation | Inactive 🡪 Active | 2×10^4^ s^-1^ | *None* | [4] |
|  | Active 🡪 Inactive | 1×10^7^ s^-1^ | Subunit Flag 4 == uThr-286 | [5, 6], this paper |
|  |  |  | Subunit Flag 3 != Fully-Bound |  |
| *Select executions:* | Active + K252a 🡪 Blocked | 1×10^9^ s^-1^ | *None* | Large value |
| 1. Ca^2+^/CaM Binding | Unbound + CaM_4_ 🡪 Initially-Bound | 1×10^8^ M^-1^s^-1^ | Subunit Flag 1 == Undocked | [1, 4] |
|  |  |  | Subunit Flag 4 != PP1-bound |  |
|  |  |  | Subunit Flag 5 == uThr-306 |  |
|  | Initially-Bound 🡪 Fully-Bound | 350 s^-1^ | Subunit Flag 2 == Active | [1] |
|  | Fully-Bound 🡪 Initially-Bound | 4×10^-3^ s^-1^ | *None* | [1] |
|  | Initially-Bound 🡪 Unbound + CaM­_4_ | 590 s^-1^ | *None* | [1, 7], this paper |
| 1. Phosphorylation (Thr-286) | uThr-286 🡪 pThr-286 | 1 s^-1^ | Subunit Flag 1 == Undocked | [8] |
|  |  |  | Neighbor Flag 1 == Undocked |  |
|  |  |  | Subunit Flag 2 == Active |  |
|  |  |  | Neighbor Flag 2 == Active |  |
|  | pThr-286 + PP1🡪 PP1-bound | 3×10^6^ M^-1^s^-1^ | Subunit Flag 3 == Unbound | Derived from [9] |
|  | PP1-bound 🡪 uThr-286 + PP1 | 2 s^-1^ | *None* | [9] |
|  | PP1-bound 🡪 pThr-286 + PP1 | 0.5 s^-1^ | *None* | Derived from [9] |
| 1. Phosphorylation (Thr-306) | uThr-306 🡪 pThr-306 | 0.02 s^-1^ | Subunit Flag 1 == Undocked | [10, 11], this paper |
|  |  |  | Subunit Flag 2 == Active |  |
|  |  |  | Subunit Flag 3 == Unbound |  |

Note: Double equal signs (==) denote the conditional “true” and the exclamation-equal sign (!=) denotes the conditional “false” statement.

**Table B. Transitions, rates parameters, conditions, and citations for the non-exclusive model.**

| Flag | State Transition | Rate | Condition (Rule) | References |
| --- | --- | --- | --- | --- |
| 1. Docking | Docked 🡪 Undocked | 0.63 s^-1^ | Subunit Flag 1 == Docked | [1-3], this paper |
|  | Undocked 🡪 Docked | 35 s^-1^ | Subunit Flag 3 == Unbound  Subunit Flag 5 == uThr-306 | [1-3], this paper |
| 1. Activation | Inactive 🡪 Active | 2×10^4^ s^-1^ | *None* | [4] |
|  | Active 🡪 Inactive | 1×10^7^ s^-1^ | Subunit Flag 4 == uThr-286 | [5, 6], this paper |
|  |  |  | Subunit Flag 3 != Fully-Bound |  |
| *Select executions:* | Active + K252a 🡪 Blocked | 1×10^9^ s^-1^ | *None* | Large value |
| 1. Ca^2+^/CaM Binding | Unbound + CaM_4_ 🡪 Initially-Bound | 1×10^8^ M^-1^s^-1^ | Subunit Flag 1 == Undocked | [1, 4] |
|  |  |  | Subunit Flag 5 == uThr-306 |  |
|  | Initially-Bound 🡪 Fully-Bound | 350 s^-1^ | Subunit Flag 2 == Active | [1] |
|  | Fully-Bound 🡪 Initially-Bound | 4×10^-3^ s^-1^ | *None* | [1] |
|  | Initially-Bound 🡪 Unbound + CaM­_4_ | 590 s^-1^ | *None* | [1, 7], this paper |
| 1. Phosphorylation (Thr-286) | uThr-286 🡪 pThr-286 | 1 s^-1^ | Subunit Flag 1 == Undocked | [8] |
|  |  |  | Neighbor Flag 1 == Undocked |  |
|  |  |  | Subunit Flag 2 == Active |  |
|  |  |  | Neighbor Flag 2 == Active |  |
|  | **pThr-286 + PP1🡪 PP1-bound** | **3×10^6^ M^-1^s^-1^** | ***None*** | Derived from [9] |
|  | PP1-bound 🡪 uThr-286 + PP1 | 2 s^-1^ | *None* | [9] |
|  | PP1-bound 🡪 pThr-286 + PP1 | 0.5 s^-1^ | *None* | Derived from [9] |
| 1. Phosphorylation (Thr-306) | uThr-306 🡪 pThr-306 | 0.02 s^-1^ | Subunit Flag 1 == Undocked | [10, 11], this paper |
|  |  |  | Subunit Flag 2 == Active |  |
|  |  |  | Subunit Flag 3 == Unbound |  |

Note: Differences in Flags 3 & 4 between Table A and Table B.

**Table S3. Transitions, rates parameters, and conditions for the exclusive 9-state-1-step model.**

| Flag | State Transition | Rate | Condition (Rule) | References |
| --- | --- | --- | --- | --- |
| 1. Ca^2+^/CaM Binding | Unbound + CaM_0_ 🡪 CaM_0_-CaMKII  Unbound + CaM_1C_ 🡪 CaM_1C_-CaMKII  Unbound + CaM_2C_ 🡪 CaM_2C_-CaMKII  Unbound + CaM_1N_ 🡪 CaM_1N_-CaMKII  Unbound + CaM_2N_ 🡪 CaM_2N_-CaMKII  Unbound + CaM_1N1C_ 🡪 CaM_1N1C_-CaMKII  Unbound + CaM_2N1C_ 🡪 CaM_2N1C_-CaMKII  Unbound + CaM_1N2C_ 🡪 CaM_1N2C_-CaMKII  Unbound + CaM_4_ 🡪 CaM_4_-CaMKII  CaM_0_-CaMKII 🡪 Unbound+CaM_0_  CaM_1C_-CaMKII 🡪 Unbound+CaM_1C_  CaM_2C_-CaMKII 🡪 Unbound+CaM_2C_  CaM_1N_-CaMKII 🡪 Unbound+CaM_1N_  CaM_2N_-CaMKII 🡪 Unbound+CaM_2N_  CaM_1N1C_-CaMKII 🡪 Unbound+CaM_1N1C_  CaM_2N1C_-CaMKII 🡪 Unbound+CaM_2N1C_  CaM_1N2C_-CaMKII 🡪 Unbound+CaM_1N2C_  CaM_4_-CaMKII 🡪 Unbound+CaM_4_  CaM_0_-CaMKII 🡪 Unbound+CaM_0_  CaM_1C_-CaMKII 🡪 Unbound+CaM_1C_  CaM_2C_-CaMKII 🡪 Unbound+CaM_2C_  CaM_1N_-CaMKII 🡪 Unbound+CaM_1N_  CaM_2N_-CaMKII 🡪 Unbound+CaM_2N_  CaM_1N1C_-CaMKII 🡪 Unbound+CaM_1N1C_  CaM_2N1C_-CaMKII 🡪 Unbound+CaM_2N1C_  CaM_1N2C_-CaMKII 🡪 Unbound+CaM_1N2C_  CaM_4_-CaMKII 🡪 Unbound+CaM_4_  Ca^2+^ + CaM_0_-CaMKII $\leftrightarrow$ CaM_1C_-CaMKII  Ca^2+^ + CaM_0_-CaMKII $\leftrightarrow$ CaM_1N_-CaMKII  Ca^2+^ + CaM_1C_-CaMKII $\leftrightarrow$ CaM_2C_-CaMKII  Ca^2+^ + CaM_1C_-CaMKII $\leftrightarrow$ CaM_1N1C_-CaMKII  Ca^2+^ + CaM_1N_-CaMKII $\leftrightarrow$ CaM_2N_-CaMKII  Ca^2+^ + CaM_1N_-CaMKII $\leftrightarrow$ CaM_1N1C_-CaMKII  Ca^2+^ + CaM_1N1C_-CaMKII $\leftrightarrow$ CaM_1N2C_-CaMKII  Ca^2+^ + CaM_2N_-CaMKII $\leftrightarrow$ CaM_2N1C_-CaMKII  Ca^2+^ + CaM_2C_-CaMKII $\leftrightarrow$ CaM_1N2C_-CaMKII  Ca^2+^ + CaM_2C_-CaMKII $\leftrightarrow$ CaM_1N2C_-CaMKII  Ca^2+^ + CaM_2N1C_-CaMKII $\leftrightarrow$ CaM_4_-CaMKII  Ca^2+^ + CaM_1N2C_-CaMKII $\leftrightarrow$ CaM_4_-CaMKII | 0.0038μM^-1^s^-1^  0.059 μM^-1^s^-1^  0.92 μM^-1^s^-1^  0.022 μM^-1^s^-1^  0.12 μM^-1^s^-1^  0.33 μM^-1^s^-1^  1.9 μM^-1^s^-1^  5.2 μM^-1^s^-1^  30 μM^-1^s^-1^  5.5 sec^-1^  6.1 sec^-1^  6.8 sec^-1^  3.1 sec^-1^  1.7 sec^-1^  3.4 sec^-1^  1.9 sec^-1^  3.8 sec^-1^  1.7 sec^-1^  5.5/1000 sec^-1^  6.1/1000 sec^-1^  6.8/1000 sec^-1^  3.1/1000 sec^-1^  1.7/1000 sec^-1^  3.4/1000 sec^-1^  1.9/1000 sec^-1^  3.8/1000 sec^-1^  1.7/1000 sec^-1^  44 μM^-1^s^-1^; 33 sec^-1^  76 μM^-1^s^-1^; 300 sec^-1^  44 μM^-1^s^-1^; 2.7 sec^-1^  76 μM^-1^s^-1^; 300 sec^-1^  76 μM^-1^s^-1^; 29.6 sec^-1^  44 μM^-1^s^-1^; 33 sec^-1^  44 μM^-1^s^-1^; 2.7 sec^-1^  44 μM^-1^s^-1^; 33 sec^-1^  76 μM^-1^s^-1^; 300 sec^-1^  76 μM^-1^s^-1^; 29.6 sec^-1^  44 μM^-1^s^-1^; 2.7 sec^-1^  76 μM^-1^s^-1^; 29.6 sec^-1^ | Flag 2 != PP1-bound  Flag 3 == uThr-306  Flag 2 != PP1-bound  Flag 3 == uThr-306  Flag 2 != PP1-bound  Flag 3 == uThr-306  Flag 2 != PP1-bound  Flag 3 == uThr-306  Flag 2 != PP1-bound  Flag 3 == uThr-306  Flag 2 != PP1-bound  Flag 3 == uThr-306  Flag 2 != PP1-bound  Flag 3 == uThr-306  Flag 2 != PP1-bound  Flag 3 == uThr-306  Flag 2 != PP1-bound  Flag 3 == uThr-306  *None*  *None*  *None*  *None*  *None*  *None*  *None*  *None*  *None*  Flag 2 == pThr-286  Flag 2 == pThr-286  Flag 2 == pThr-286  Flag 2 == pThr-286  Flag 2 == pThr-286  Flag 2 == pThr-286  Flag 2 == pThr-286  Flag 2 == pThr-286  Flag 2 == pThr-286  *None*  *None*  *None*  *None*  *None*  *None*  *None*  *None*  *None*  *None*  *None*  *None* | [12]  [12]  [12]  [12]  [12]  [12]  [12]  [12]  [12]  [12]  [12]  [12]  [12]  [12]  [12]  [12]  [12]  [12]  [13]  [13]  [13]  [13]  [13]  [13]  [13]  [13]  [13]  [12]  [12]  [12]  [12]  [12]  [12]  [12]  [12]  [12]  [12]  [12]  [12] |
| 1. Phosphorylation (Thr-286) | uThr-286 🡪 pThr-286 | 1 s^-1^ | Flag 1 != Unbound | [8] |
|  | pThr-286 + PP1🡪 PP1-bound | 3×10^6^ M^-1^s^-1^ | Flag 1 == Unbound | [9] |
|  | PP1-bound 🡪 uThr-286 + PP1 | 2 s^-1^ | *None* | [9] |
|  | PP1-bound 🡪 pThr-286 + PP1 | 0.5 s^-1^ | *None* | [9] |
| 1. Phosphorylation (Thr-306) | uThr-306 🡪 pThr-306 | 0.02 s^-1^ | Flag 1 != Unbound | [10, 11], this paper |

Note: Parameters for Ca^2+^-CaM binding (for CaM not bound to CaMKII) are given in [12] and [14].

Figure A. Model validation at long Ca^2+^ peak widths.


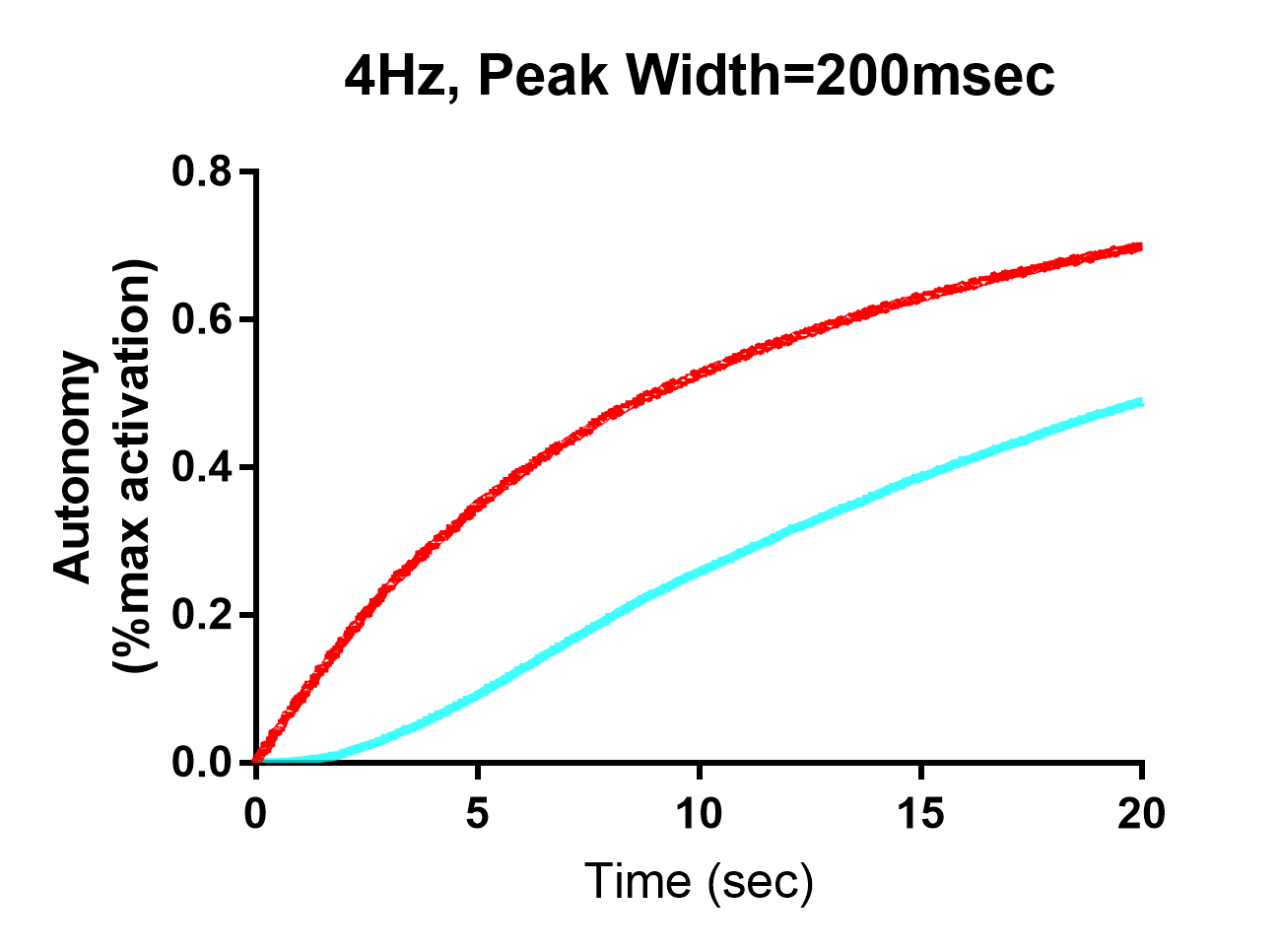


Figure A. Here, we simulate the 2-state-2-step model under identical conditions to those in Figure 3B in De Koninck and Schulman (*Science*, 1998) [15]. Specifically, the model is continuously stimulated by 4Hz Ca^2+^/CaM with peak widths of 200msec and in the absence of PP. Because the CaMKII subunit concentration is not reported the Schulman study, we use a [CaM] to [CaMKII subunit] ratio of 1:1. We monitor active (red) and pThr-286 (cyan) CaMKII subunits relative to the total concentration of CaMKII subunits (18.24 μM). At t=20sec, our model predicts 50% maximum CaMKII subunit phosphorylation, in agreement with the De Koninck experimental results.

Figure B. CaM activation/inactivation parameters as a function of time.


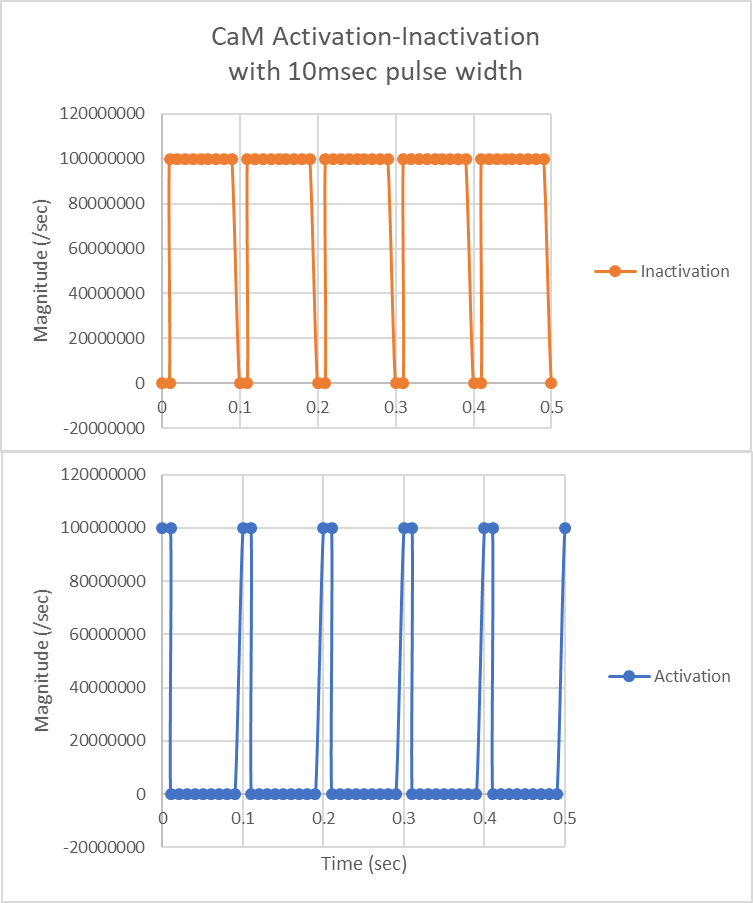


Figure B. Representative traces of the parameters “inactivation” (orange) and “activation” (blue), which in the frequency-dependent simulations vary as a function of time, for the complete duration of the simulation, to force CaM into a fully inactive or active state. Here, we show activation pulse widths at our default value of 10msec.

Text A. Necklace Number Quantification of CaMKII Combinatorial Explosion

In the absence of rules defining the conditions for subunit state transition, CaMKII subunit states are independent, causing combinatorial explosion. To quantify the combinatorial explosion, consider each of the five flags denoting CaMKII subunit states in our model. The docking flag can have one of **two** states. The activation flag can have one of **two** states. The CaM-binding flag can have one of **three** states. The Thr-286 flag can have one of **three** states. Finally, the Thr-306 flag can have one of **two** states. If each flag state is independent, there is a total of **72** possible state combinations that a single subunit can exhibit.

Next, we consider a single ring of six subunits, noting the rotational symmetry. Leveraging this symmetry, we refer to so-called “necklace numbers”. Using necklace numbers, the number of fixed arrangements of beads on the necklace is defined as:

$$N\left( n,a \right)=\frac{1}{n}\sum_{i=1}^{v(n)} \phi\left( d_{i} \right) a^{n/d_{i}}$$

Where n is the length of the necklace and a is the number of bead types. Phi is Euler’s totient function, and d are divisors of n. Thus, for a single CaMKII ring in which n=6 and a=72, the possible number of state combinations is: 23219075544.

To count possible states for a single holoenzyme, we again use necklace numbers. (Squaring the number calculated for a single CaMKII ring fails to account for rare cases in which both rings exhibit identical states.) Thus, we calculate the number of states for the necklace with n=2 and a=23219075544. With this calculation, we determine that in the absence of rules, a twelve-subunit CaMKII holoenzyme as modeled in this paper has **269562734570598985740** possible states.

We emphasize that this value is our estimation. We also acknowledge David Sterratt for his suggestion to use necklace numbers in this calculation.


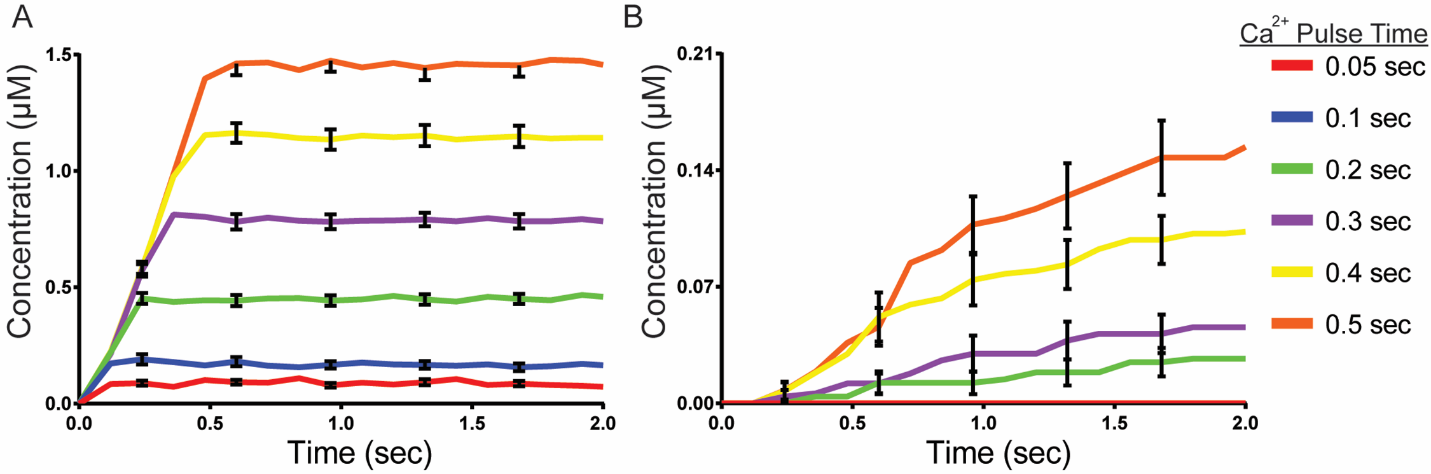
Figure C. Re-creation of Figure 4, showing SEM error bars where all traces are the average of N=50 executions.

Text B. Exploration of Multiple Degrees of Freedom for Autophosphorylation

To explore the dependence of CaMKII pThr-286 patterns on varying mechanisms of autophosphorylation, we present alternative versions of our model in which autophosphorylation may occur in multiple directions, intra- and/or trans-ring. In these higher-degrees of freedom (DoF) models, autophosphorylation may occur in two directions (2 DoF) or three directions (3 DoF).


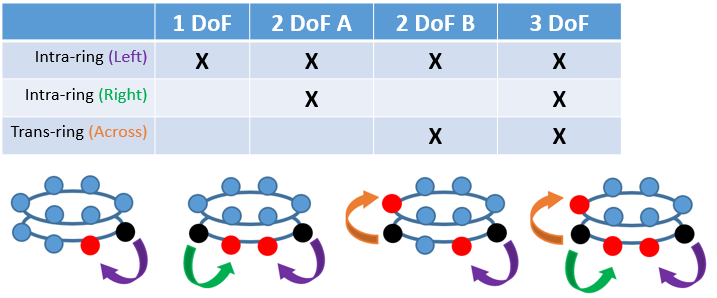


Fig D. Schematic of higher-DoF autophosphorylation of CaMKII subunits (blue). Black circles are the enzymes and red circles are the substrate subunits participating in an autophosphorylation.

We hypothesized that higher DoF’s could elicit higher or more robust levels of CaMKII autophosphorylation in the presence of phosphatase. Therefore, in Figure E below, we monitor pThr-286 in each of the DoF models as a function of time. Each model is subjected to saturating levels of Ca^2+^/CaM for 20 sec, after which the stimulating Ca^2+^/CaM is removed. The results in Figure E indicate that a higher DoF may allow for more rapid formation of pThr-286. For example, the slope of the 3-DoF trace (pink) at t=10sec is noticeably greater than that for the 1-DoF trace (dark blue). Also, following stimulation (after t=20sec) de-phosphorylation rate seems to be independent of DoF. We note that for the models as-simulated and as-parameterized in Figure E, the differences in output between each DoF model may not be experimentally distinguishable.


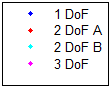


Figure E. Comparing pThr-286 formation for various model DoF for autophosphorylation. For all simulations, holoenzymes were stimulated in the presence of phosphatase with a saturating level of Ca^2+^/CaM for 20sec, after which Ca^2+^/CaM was removed. Each trace is the average of N=50 executions.

It is also interesting to consider how a higher DoF could accelerate and/or provide redundant paths by which information (e.g. autophosphorylations) flows from one end of a holoenzyme to the opposite. Indeed, intra-holoenzyme information transfer could impact how CaMKII operates as a simultaneous signaling and structural protein, such as when one subunit interacts with Ca^2+^/CaM while a neighboring subunit binds to actin [16].

To explore the impact of higher DoF autophosphorylation on intra-holoenzyme information transfer, we use the MCell rule-based modeling syntax to monitor individual subunits on an individual holoenzyme. Monitoring individual subunits allows us to identify CaMKII autophosphorylation “chains”, defined as a series of consecutive autophosphorylated subunit neighbors. In Figure F, we monitor individual CaMKII subunit autophosphorylations over time, in the continuous presence of saturating Ca^2+^/CaM and phosphatase, for various DoFs. In Figure G, we quantify the dependence of autophosphorylation chain formation on DoF number. Increasing DoF increases the lifetime of autophosphorylation chains (of any length), albeit marginally leading us to suspect experimental measurements would be inconclusive.


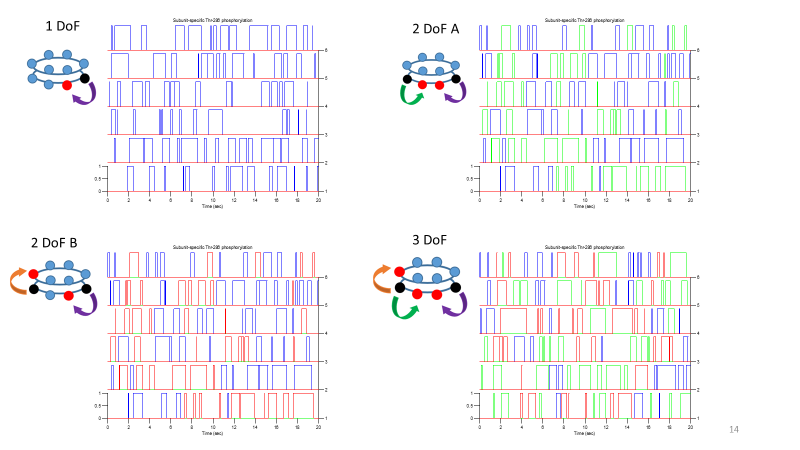


Figure F. Monitoring individual CaMKII subunits at distinct autophosphorylation DoF. In each panel, the horizonal axis is time (up to 20 sec). Each row pertains to one of six subunits in a single holoenzyme ring, and each trace is a binary representation of whether that subunit is pThr-286 (one) or uThr-286 (zero). Depending on the number of DoF, autophosphorylations may proceed clockwise (blue), counter-clockwise (green), and/or trans-ring (red).

Figure G. pThr-286 chain occurrence increases with increasing DoF. Here, we monitored a single CaMKII holoenzyme over 2min. Each bar denotes the fraction of time that a chain of length n occurs for that number of DoF. Each bar is the average of N=50 executions.

Movie A.

Please see this link - <https://youtu.be/lUSuv2mWa5g>.

Text C. Discussion of putative PP1-CaMKII binding site.

Many PP1-binding proteins contain a variable binding motif commonly called the RVxF binding motif [17]. However, no such motif is seen in the alpha-CaMKII subunit amino acid sequence. Furthermore, no structure for the PP1-CaMKII interaction exists, in part because apo-PP1 is unstable in solution [18]. We can instead refer to a crystal structure published by Ragusa *et al*., which shows PP1 binding the neuronal regulatory protein spinophilin (PDB 3EGG) [19].

The 3EGG crystal structure shows an interaction with PP1 spanning the spinophilin residues 417-494. This interaction involves 77 residues (~100 Å, see Figure H), which are necessary and sufficient for PP1 to dephosphorylate spinophilin [20]. This interaction distance is longer than the CaMKII regulatory domain and CaM-binding footprint. Indeed, the CaMKII regulatory domain is only about 39 Å (as in PDB 3SOA, partially shown in Figure I).


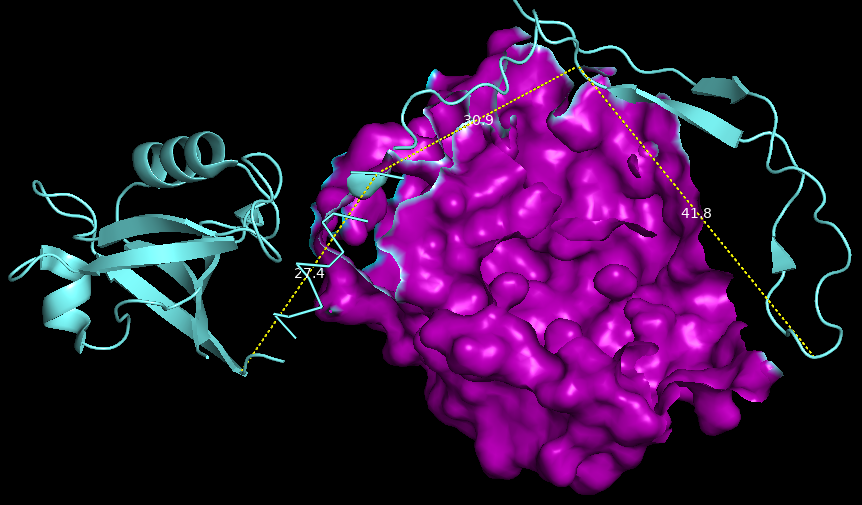


Figure H. Visualization of the spinophilin (cyan) and PP1 (purple) binding interaction (PDB: 3EGG). Some of the PP1 surface is digitally removed to help visualize the dotted lines, which together approximate the distance spanned by spinophilin residues 417-494.


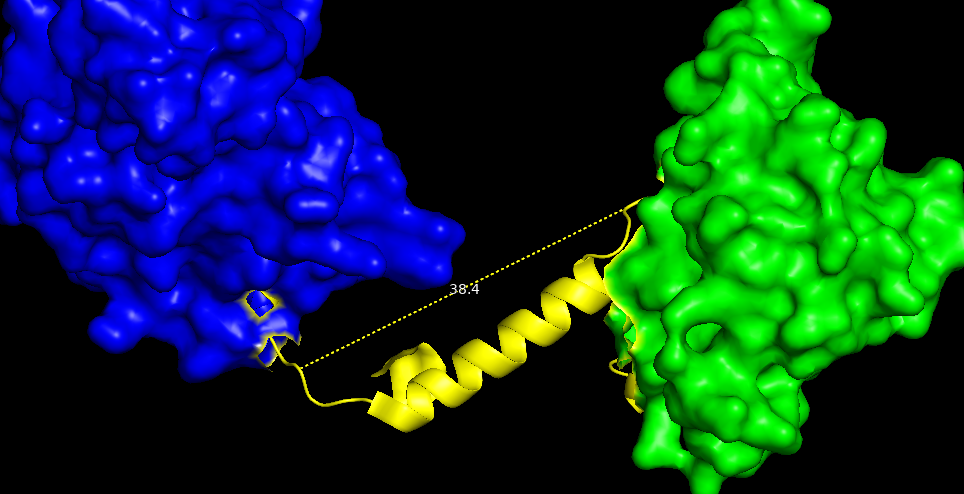


Figure I. Visualization of the CaMKII regulatory domain (yellow) (PDB: 3SOA). The CaMKII hub domain (green) and kinase domain (blue) are also shown. Dotted line measures from residues 280-306.

Note also that the typical PP1 binding motif interacts with PP1 at a site roughly 20 Å from the PP1 active site [20]. Thus, if the PP1 binding footprint does not contain T286, then the furthest CaMKII residue the PP1 footprint could likely be (on the hub domain side of T286) is residue 301, well within the CaM-binding footprint (see Figure J). Admittedly, the furthest likely PP1-binding residue on the kinase domain side of T286 is difficult to ascertain from PDB 3SOA. Still, we believe there is strong evidence that CaM structurally excludes or limits PP1 from binding and/or catalyzing de-phosphorylation at T286.


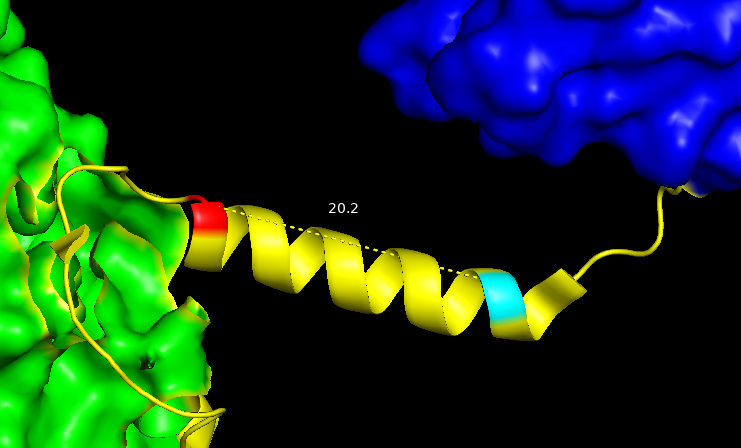


Figure J. Visualization of the furthest likely residue (G301; red) of PP1-binding on the hub domain (green) side of T286 (cyan) (PDB: 3SOA). Dotted line measures from residues 286-301.

Figure K. Major Ca^2+^/CaM-CaMKII states following Ca^2+^ bolus in the 9-state-1-step model


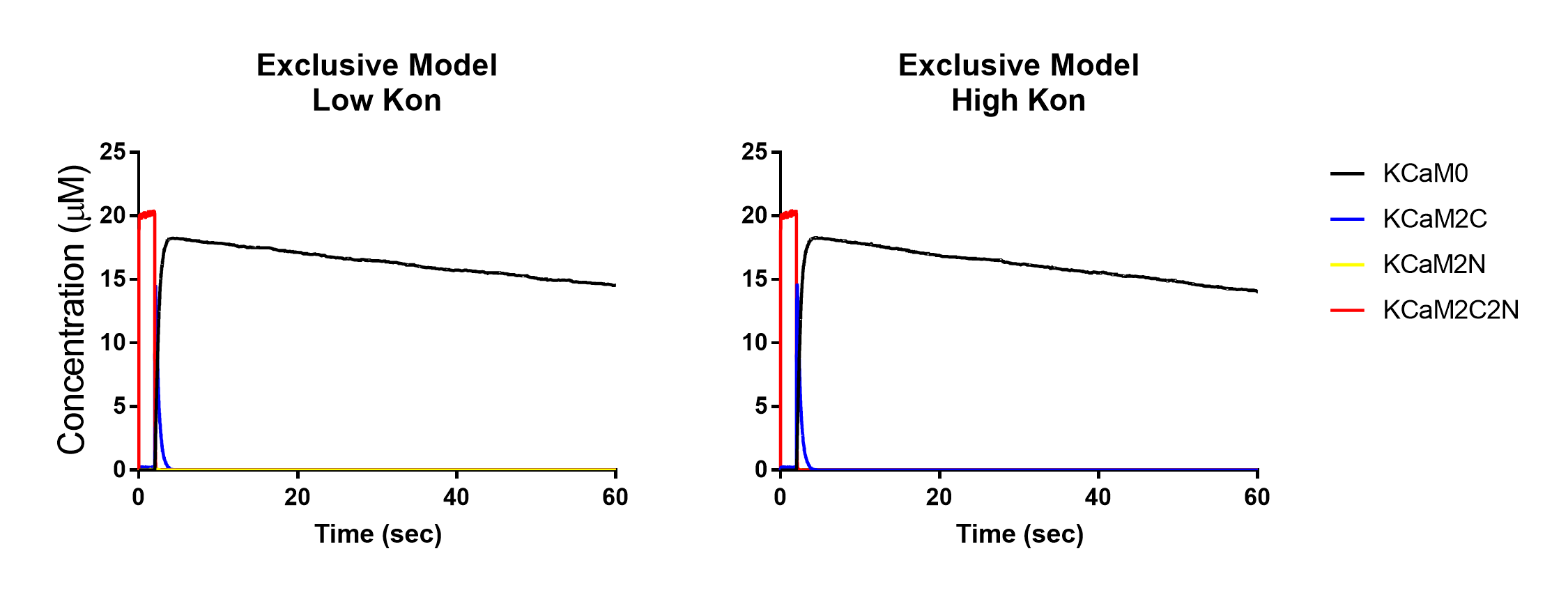


Figure K. Bound Ca^2+^/CaM states in response to 2 sec Ca^2+^ bolus as-implemented in Fig 7. We monitor the CaMKII-bound Ca^2+^/CaM states apo-CaM (black), CaM_2C_ (blue), CaM_2N_ (yellow), and CaM_4_ (or CaM_2C2N_, red) in the PP1-exclusive 9-state-1-step model under low (left) and high (right) association rate parameters for PP1-CaMKII binding. Each trace is the average of N=20 executions.

Figure L. Ca^2+^/CaM-CaMKII states with dynamic Ca^2+^ stimulation and the 9-state-1-step model


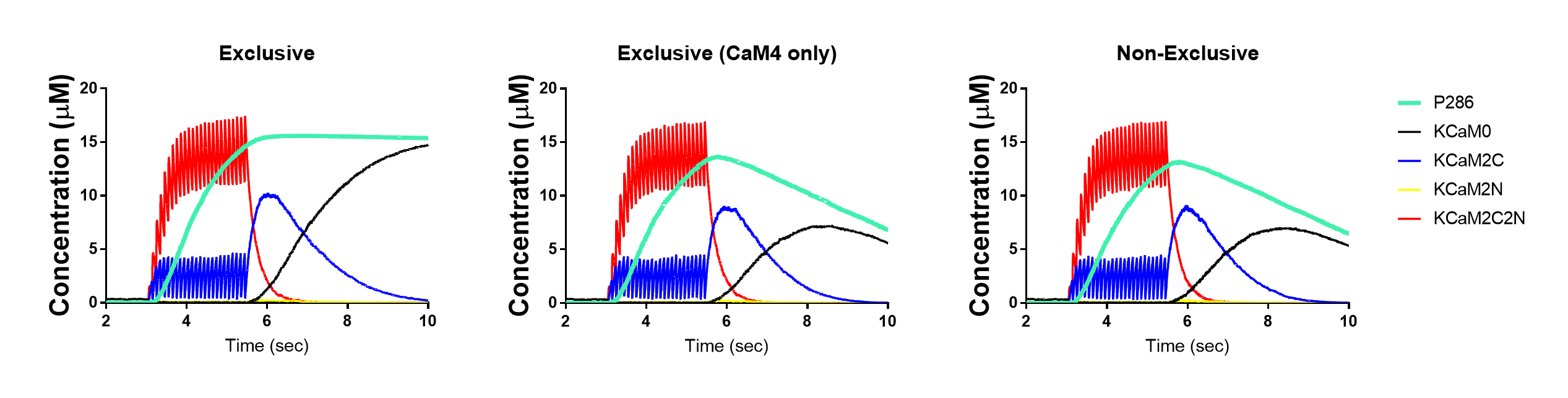


Figure L. CaMKII-bound Ca^2+^/CaM states and pThr-286 subunits (cyan) in response to 10Hz Ca^2+^ flux. Peak pThr-286 levels at t=6 sec are highest in the exclusive model (15.4 μM). When only CaM_4_ is allowed to exclude PP1-binding, pThr-286 levels at t=6 sec are 13.5 μM, whereas pThr-286 levels at the same time point in the non-exclusive model are 13 μM. Also, at t=10 sec pThr-286 levels in the non-exclusive model are lower (6.4μM) than when only CaM_4_ excludes PP1-binding (7μM), seemingly due to heightened CaM_2C_ levels immediately following termination of Ca^2+^ flux. Altogether, it seems that during and immediately following dynamic Ca^2+^ flux, CaM_2C_ levels may be non-negligible. CaM_2C_, in addition to apo-CaM or CaM_4_, could sometimes significantly regulate pThr-286 levels via CaM-dependent PP1 exclusion. Because this is the 9-state-1-step model, each trace is the average of N=20 executions.

References (Supplement)

1. Torok K, Tzortzopoulos A, Grabarek Z, Best SL, Thorogate R. Dual effect of ATP in the activation mechanism of brain Ca2+/calmodulin-dependent protein kinase II by Ca2+/calmodulin. Biochemistry. 2001;40(49):14878-90. doi: 10.1021/bi010920+.

2. Tzortzopoulos A, Torok K. Mechanism of the T286A-mutant alpha CaMKII interactions with Ca2+/calmodulin and ATP. Biochemistry. 2004;43(21):6404-14. doi: 10.1021/bi036224m.

3. Chao LH, Stratton MM, Lee IH, Rosenberg OS, Levitz J, Mandell DJ, et al. A Mechanism for Tunable Autoinhibition in the Structure of a Human Ca2+/Calmodulin-Dependent Kinase II Holoenzyme. Cell. 2011;146(5):732-45. doi: 10.1016/j.cell.2011.07.038.

4. Stefan MI, Marshall DP, Le Novere N. Structural analysis and stochastic modelling suggest a mechanism for calmodulin trapping by CaMKII. PLoS One. 2012;7(1):14. doi: 10.1371/journal.pone.0029406.

5. Hoffman L, Chandrasekar A, Wang X, Putkey JA, Waxham MN. Neurogranin alters the structure and calcium binding properties of calmodulin. J Biol Chem. 2014;289(21):14644-55. doi: 10.1074/jbc.M114.560656.

6. Hoffman L, Stein RA, Colbran RJ, McHaourab HS. Conformational changes underlying calcium/calmodulin-dependent protein kinase II activation. Embo J. 2011;30(7):1251-62. doi: 10.1038/emboj.2011.40.

7. Tse JKY, Giannetti AM, Bradshaw JM. Thermodynamics of calmodulin trapping by Ca2+/calmodulin-dependent protein kinase II: Subpicomolar K-d determined using competition titration calorimetry. Biochemistry. 2007;46(13):4017-27. doi: 10.1021/bi700013y.

8. Shifman JM, Choi MH, Mihalas S, Mayo SL, Kennedy MB. Ca2+/calmodulin-dependent protein kinase II (CaMKII) is activated by calmodulin with two bound calciums. Proc Natl Acad Sci U S A. 2006;103(38):13968-73. doi: 10.1073/pnas.0606433103.

9. Zhabotinsky AM. Bistability in the Ca2+/calmodulin-dependent protein kinase-phosphatase system. Biophys J. 2000;79(5):2211-21. doi: 10.1016/s0006-3495(00)76469-1.

10. Lucic V, Greif GJ, Kennedy MB. Detailed state model of CaMKII activation and autophosphorylation. Eur Biophys J Biophys Lett. 2008;38(1):83-98. doi: 10.1007/s00249-008-0362-4.

11. Payne ME, Fong YL, Ono T, Colbran RJ, Kemp BE, Soderling TR, et al. Calcium calmodulin-dependent protein kinase-II - characterization of distinct calmodulin binding and inhibitory domains. J Biol Chem. 1988;263(15):7190-5.

12. Pepke S, Kinzer-Ursem T, Mihalas S, Kennedy MB. A dynamic model of interactions of Ca2+, calmodulin, and catalytic subunits of Ca2+/calmodulin-dependent protein kinase II. PLoS Comput Biol. 2010;6(2):15. doi: 10.1371/journal.pcbi.1000675.

13. Meyer T, Hanson PI, Stryer L, Schulman H. Calmodulin trapping by calcium-calmodulin dependent protein-kinase. Science. 1992;256(5060):1199-202. doi: 10.1126/science.256.5060.1199.

14. Romano DR, Pharris MC, Patel NM, Kinzer-Ursem TL. Competitive tuning: Competition's role in setting the frequency-dependence of Ca2+-dependent proteins. PLoS Comput Biol. 2017;13(11):26. doi: 10.1371/journal.pcbi.1005820.

15. De Koninck P, Schulman H. Sensitivity of CaM kinase II to the frequency of Ca2+ oscillations. Science. 1998;279(5348):227-30. doi: 10.1126/science.279.5348.227.

16. Lisman JE, Zhabotinsky AM. A model of synaptic memory: A CaMKII/PP1 switch that potentiates transmission by organizing an AMPA receptor anchoring assembly. Neuron. 2001;31(2):191-201. doi: 10.1016/s0896-6273(01)00364-6.

17. Cohen PTW. Protein phosphatase 1 - targeted in many directions. J Cell Sci. 2002;115(2):241-56. PubMed PMID: WOS:000173768800002.

18. Kelker MS, Page R, Peti W. Crystal structures of protein phosphatase-1 bound to nodularin-r and tautomycin: a novel scaffold for structure-based drug design of serine/threonine phosphatase inhibitors. J Mol Biol. 2009;385(1):11-21. doi: 10.1016/j.jmb.2008.10.053.

19. Ragusa MJ, Dancheck B, Critton DA, Nairn AC, Page R, Peti W. Spinophilin directs protein phosphatase 1 specificity by blocking substrate binding sites. Nat Struct Mol Biol. 2010;17(4):459-U100. doi: 10.1038/nsmb.1786.

20. Hsieh-Wilson LC, Allen PB, Watanabe T, Nairn AC, Greengard P. Characterization of the neuronal targeting protein spinophilin and its interactions with protein phosphatase-1. Biochemistry. 1999;38(14):4365-73. doi: 10.1021/bi982900m.
